# Validation of a Rapid Rabies Diagnostic Tool for Field Surveillance in Developing Countries

**DOI:** 10.1371/journal.pntd.0005010

**Published:** 2016-10-05

**Authors:** Monique Léchenne, Kemdongarti Naïssengar, Anthony Lepelletier, Idriss Oumar Alfaroukh, Hervé Bourhy, Jakob Zinsstag, Laurent Dacheux

**Affiliations:** 1 Swiss Tropical and Public Health Institute, Basel, Switzerland; 2 University of Basel, Basel, Switzerland; 3 Institut de Recherche en Elevage pour le Developpement, Farcha, N’Djamena, Chad; 4 Institut Pasteur, Unit Lyssavirus Dynamics and Host Adaptation, National Reference Center for Rabies and WHO Collaborating Centre for Reference and Research on Rabies, Paris, France; Wistar Institute, UNITED STATES

## Abstract

**Background:**

One root cause of the neglect of rabies is the lack of adequate diagnostic tests in the context of low income countries. A rapid, performance friendly and low cost method to detect rabies virus (RABV) in brain samples will contribute positively to surveillance and consequently to accurate data reporting, which is presently missing in the majority of rabies endemic countries.

**Methodology/Principal findings:**

We evaluated a rapid immunodiagnostic test (RIDT) in comparison with the standard fluorescent antibody test (FAT) and confirmed the detection of the viral RNA by real time reverse transcription polymerase chain reaction (RT-qPCR). Our analysis is a multicentre approach to validate the performance of the RIDT in both a field laboratory (N’Djamena, Chad) and an international reference laboratory (Institut Pasteur, Paris, France). In the field laboratory, 48 samples from dogs were tested and in the reference laboratory setting, a total of 73 samples was tested, representing a wide diversity of RABV in terms of animal species tested (13 different species), geographical origin of isolates with special emphasis on Africa, and different phylogenetic clades. Under reference laboratory conditions, specificity was 93.3% and sensitivity was 95.3% compared to the gold standard FAT test. Under field laboratory conditions, the RIDT yielded a higher reliability than the FAT test particularly on fresh and decomposed samples. Viral RNA was later extracted directly from the test filter paper and further used successfully for sequencing and genotyping.

**Conclusion/Significance:**

The RIDT shows excellent performance qualities both in regard to user friendliness and reliability of the result. In addition, the test cassettes can be used as a vehicle to ship viral RNA to reference laboratories for further laboratory confirmation of the diagnosis and for epidemiological investigations using nucleotide sequencing. The potential for satisfactory use in remote locations is therefore very high to improve the global knowledge of rabies epidemiology. However, we suggest some changes to the protocol, as well as careful further validation, before promotion and wider use.

## Author Summary

The high fatality and burden of rabies stands in contrast to the very low performance of laboratory-based surveillance in resource-challenged countries. The absence of reliable human and animal rabies incidence data ultimately result in neglect of disease prevention and control and the perpetuation of RABV transmission despite the existence of powerful management tools. Rapid, easy to perform rabies diagnostic tests that do not require expensive equipment or special storage conditions, which can be reliably performed by trained ordinary veterinary professionals, are needed urgently for use in low income countries. Such novel methods will help to accurately assess the global rabies burden and are necessary to monitor rabies control and elimination. The present study evaluates the performance and reliability of a rapid, easy to use rabies diagnostic tool. Overall, the validated test was in high accordance with the standard reference method for the detection of RABV by immunofluorescence microscopy and showed even higher reliability when applied in resource poor laboratory conditions. The obtained results support the high potential for the use of this test in the field but suggest a change of the original technical protocol and a need for wider validation.

## Introduction

Rabies is a viral zoonotic encephalomyelitis transmitted to humans after exposure to infected mammals, mainly dogs, through bites, scratches or licks on damaged skin or mucous membranes. This disease still continues to represent a public health concern worldwide, with an estimate of 60,000 human deaths per year, mainly in low income countries. Because of limited control measures in many countries and a lack of governmental concern, rabies remains a neglected tropical disease. This neglect is especially deplorable given the entirely preventable nature of the disease through vaccination of dogs and timely adherence to post-exposure prophylaxis (PEP) of exposed victims [1, 2]. In this way, human deaths due to this zoonotic disease could be reduced by over 95% [3, 4].

Lack of surveillance represents one major element of negligence, leading to missing data on disease incidence, imprecise estimates of the economic impact and a general underestimation of the true worldwide burden of rabies [4, 5, 6, 7]. This means that advocacy for rabies control cannot be supported with solid evidence and the necessity for action is not perceived at the decision maker level, for instance governmental authorities [8]. Poor surveillance is primarily a result of lack of political commitment and resource attribution for the control of rabies, and thus a cycle of neglect perpetuates. The vicious cycle is reinforced by the disregard of disease control in domestic dogs, which constitute negligible economic value and the fact that rabies affects largely marginalized communities with difficult access to healthcare. Deficiencies in basic healthcare do not only contribute to hinder access to PEP but also lead to the misdiagnosis of rabies in the face of other causes of encephalitis, such as cerebral malaria, as reported in Malawi [9]. Surveillance is fundamental to accurate burden of disease measures, for advocacy of disease control and also a prerequisite for disease elimination [10, 11]. Currently, rabies is believed to be underreported at the extent of 1:60 in humans and this rate could even be much higher for animal rabies incidence [6].

One possible point of leverage to break the cycle of underreporting and neglect is the reinforcement and simplification of diagnostic capacities and tools. Infrastructures required for the current standard diagnostic tests are expensive, and their methodologies and interpretation need thoroughly experienced personnel. Antigen detection of RABV using the direct fluorescent antibody test (FAT) is the World Health Organization (WHO) and World Organisation for Animal Health (OIE) reference test [6, 12, 13] and is routinely performed in many developed countries. However, it is difficult to establish in developing countries because fluorescence microscopes are expensive and the required maintenance is demanding. Also, the immunofluorescence conjugate necessary for the test is costly and has to be transported and stored refrigerated. Finally, accurate reading of the test needs stringent quality control of the test performance and very experienced personnel. Similar constraints are encountered with the direct rapid immunohistochemical test (DRIT) [14, 15]. Although the DRIT can be read using a light microscope, the test methodology requires a meticulous protocol which currently lacks commercialized biotinylated anti-rabies antibodies and has to be carried out by trained personnel. For rabies diagnosis however, a simpler field test is desirable for various reasons. In the current situation the benefits of rabies diagnosis are not well perceived by the public and rabies suspicious animals are often killed immediately and rapidly disposed [16]. Also, a short time lag between suspicion and confirmation of a rabies case is important for early adherence to PEP or the cost savings in case of a negative diagnostic result. Finally, transport of samples over long distances in climatically warm settings increases the risk of poor sample quality, which adversely affects FAT test results [17]. Proximity to the public through decentralized laboratory facilities is therefore vital for good sample quality, as well as rapid detection and response.

A rapid immunodiagnostic test (RIDT) based on the lateral flow principle was first described and evaluated in 2007 on a limited panel of RABV samples [18]. The same study reported on the detection limit and potential risk of cross reactivity. Further laboratory evaluation was conducted more recently on the use of this RIDT for the detection of RABV circulating in Europe, and extended to the detection of other species of lyssaviruses [19, 20]. Both studies showed positive results regarding sensitivity and specificity of such tests compared to the FAT. To date, only two studies have been conducted under field conditions, both suggesting positive results for the use of RIDT [21, 22].

In our study, we evaluated the practicability and the performance of this RDIT identified as Anigen Rapid Rabies Test (Anigen test) (Bionote Inc.) in different settings: under field conditions with its application to the surveillance of rabid animals in N’Djamena, Chad and in laboratory settings with a panel of selected RABV isolates. Lastly, we evaluated this tool for a novel application in rabies surveillance, with its use as a vehicle for viral RNA storage and conservation, and demonstrated that recovery and detection of RNA present on the strip of positive samples was possible. The Anigen test appears as a promising tool for the *post-mortem* diagnosis of animal rabies, and the molecular detection and genotyping of positive test strips.

## Materials and Methods

### Implementation of the Anigen test in the field

During June 2012, the RIDT Anigen test, a chromatographic immunoassay-based on lateral flow technology manufactured by BioNote, Inc (Gyeongi-do, Republic of Korea) [23], was added into the routine diagnostic procedure of the rabies laboratory of the Institut de Recherche en Elevage pour le Développement (IRED) in N’Djamena, Chad. It was utilized in parallel with the FAT test, which had been used since 2001. Rabies-suspect animals were presented to the IRED by their owners or by the bite victim. No active surveillance was initiated throughout the study. However, awareness was intensified prior to the study period, during May 2012, by a poster campaign sensitizing the public in N’Djamena to seek medical treatment after a dog bite and to send the biting animal to the IRED in case of rabies suspicion.

### Description of samples and isolates

For the validation of the Anigen test in the field, diagnostic results from June 2012 to February 2015 were included. Only samples originating from dogs were considered for inclusion according to the manufacturer’s recommendation [23]. During the 33 months of the study period, a total of 49 rabies -suspect dog heads were submitted to IRED for diagnostic testing. The origin of the samples is detailed in Table 1. Most were in fresh condition on arrival and upon testing (85%, n = 42). Five of the samples were decomposed, while in one case, the sample quality was not noted. Only one sample was so decomposed that it was impossible to analyse and was excluded from the study. The final sample size of field isolates at IRED was 48 (Table 1).

**Table 1:** Description of samples tested in Chad and results obtained after FAT, RIDT and RT-qPCR on FTA papers. All specimens correspond to original brain samples collected from dogs.

Identification	Sampling date	Origin	Condition	FAT Result	RIDT Result	RT-qPCR Result
342	06.06.2012	N'Djamena	Fresh	Neg	Neg	Neg
343	21.06.2012	N'Djamena	Decomposed	Pos	Neg	Neg
344	22.06.2012	N'Djamena	Fresh	Pos	Pos	Pos
345	28.06.2012	N'Djamena	Fresh	Pos	Pos	Pos
346	05.07.2012	N'Djamena	Fresh	Pos	Pos	Pos
347	13.07.2012	Koundoul	Fresh	Pos	Pos	Pos
348	20.07.2012	N'Djamena	Fresh	Pos	Pos	Pos
349	31.07.2012	N'Djamena	Fresh	Pos	Pos	Pos
350	08.08.2012	N'Djamena	Fresh	Pos	Pos	Pos
352	25.08.2012	N'Djamena	Fresh	Pos	Pos	Pos
354	29.08.2012	N'Djamena	Fresh	Pos	Pos	Pos
355	11.09.2012	N'Djamena	Fresh	Pos	Pos	Pos
356	12.09.2012	N'Djamena	Fresh	Pos	Pos	Pos
357	20.09.2012	N'Djamena	Fresh	Pos	Pos	Pos
358	25.09.2012	N'Djamena	Fresh	Pos	Pos	Pos
359	28.09.2012	unknown	Fresh	Pos	Pos	Pos
360	01.10.2012	N'Djamena	Fresh	Neg	Neg	Neg
361	26.10.2012	N'Djamena	Fresh	Pos	Pos	Pos
362	24.12.2024	unknown	Unknown	Pos	Neg	Neg
363	19.11.2012	N'Djamena	Fresh	Pos	Pos	Pos
364	03.12.2012	N'Djamena	Fresh	Pos	Pos	Pos
365	05.12.2012	N'Djamena	Fresh	Neg	Neg	Neg
366	06.12.2012	N'Djamena	Fresh	Pos	Pos	Pos
367	10.01.2013	Moundou	Fresh	Pos	Pos	Pos
368	11.01.2013	N'Djamena	Fresh	Pos	Pos	Pos
369	08.02.2013	N'Djamena	Fresh	Pos	Pos	Pos
371	25.03.2013	Koundoul	Fresh	Pos	Pos	Pos
372	29.05.2013	N'Djamena	Fresh	Pos	Pos	Pos
373	17.06.2013	Koundoul	Fresh	Pos	Pos	Pos
379	12.11.2013	N'Djamena	Fresh	Pos	Pos	Pos
380	12.12.2013	N'Djamena	Fresh	Neg	Neg	Neg
381	31.01.2014	N'Djamena	Fresh	Pos	Pos	Pos
383	16.04.2014	N'Djamena	Fresh	Neg	Neg	ND
384	29.04.2014	N'Djamena	Fresh	Neg	Neg	ND
386	29.04.2014	Guelendeng	Fresh	Neg	Neg	ND
389	28.05.2014	N'Djamena	Decomposed	Imp	Neg	Neg
390	06.06.2014	N'Djamena	Fresh	Neg	Neg	ND
392	23.06.2014	N'Djamena	Decomposed	Neg	Neg	ND
393	14.07.2014	Koundoul	Fresh	Neg	Neg	ND
394	18.08.2014	Loumia	Fresh	Pos	Pos	Pos
395	21.08.2014	Chawaye	Fresh	Pos	Pos	Pos
400	13.10.2014	N'Djamena	Fresh	Neg	Neg	ND
401	14.10.2014	N'Djamena	Fresh	Pos	Pos	Pos
403	09.12.2014	Loumia	Fresh	Pos	Pos	Pos
405	27.01.2015	N'Djamena	Fresh	Pos	Pos	Pos
406	05.02.2015	Djermaya	Decomposed	Pos	Pos	ND
407	11.02.2015	Dourbali	Decomposed	Pos	Pos	Pos
408	13.02.2015	N'Djamena	Fresh	Pos	Pos	Pos

Pos: positive, Neg: negative, Imp: impossible, ND: not done.

The Anigen test was further validated at the National Reference Centre (NRC-R) and WHO Collaborating Center for Rabies at the Institut Pasteur in Paris, France, on 73 samples selected from the collections housed in both of these centers, from 12 different species originating from various countries and belonging to different phylogenetic clades (S1 Table). All these samples were previously analysed by FAT. Thirty of them were negative and the remaining 43 were positive. The positive samples represented a large diversity of RABV. All these 73 samples were stored at -80°C for archive before analysis.

In addition, the limit of detection of the RIDT was evaluated at NRC-R using a panel of 8 different isolates of RABV adapted and amplified on baby hamster kidney cells (BSR cells). Viral suspensions were titrated on the same cells using 5-fold serial dilutions in cell culture medium and expressed as fluorescent focus units per mL (FFU/mL). For the RIDT evaluation, titrated RABV suspensions were first tested at several concentrations using the buffer available from the RIDT kit as a diluent.

### Description of the FAT methodology

The FAT, the gold standard technique for *post-mortem* diagnosis of rabies [12] was performed at the NRC-R under quality assurance (accreditation ISO/IEC 17025), as previously described [13]. In the rabies laboratory of N’Djamena, the FAT was performed with some deviations regarding the standard procedure: lack of positive and negative control samples inclusion, absence of routine quality assessments, and storage of the immunofluorescent conjugate past the expiration date. In this setting, two microscopic slides were prepared, with two brain impressions per slide. If no viral characteristic fluorescent inclusions were observed on all four impressions, the sample was considered negative. Doubtful results were declared positive due to the potential fatal consequences of a false negative result for the bite victims. However, due to some deviations regarding the standard procedure, it was not possible to consider FAT performed ad IRED as the gold standard for the specificity and sensitivity analysis.

### Description of the RIDT Anigen test methodology

The Anigen test is a simple and rapid diagnostic tool, presenting as an all-in-one included kit. Once the brain is extracted, it is used without additional material and equipment except for one dilution step requiring an additional vial of phosphate-buffered saline (PBS) prepared according to the manufacturer’s recommendation. However, for our study, we omitted the first dilution step, only using the vial with buffer provided by the kit to simplify the test procedure in view of future application under realistic field conditions. The same procedure was used for the Anigen test at NRC-R and at IRED. If it was possible to anatomically identify the regions of the brain in a sample, the test was performed with a small section of the brainstem (approximately 0.1 g), otherwise the same amount of material was taken from different parts of decomposed brain samples. The brain sample was mixed directly in the tube containing the buffer with the swab, all included in the kit, for about one minute, until most of the brain material was well dissolved and then put on to the test plate using the transfer pipette provided in the kit. Four drops were deposited on the strip (corresponding to nearly 100 μL). The test could be interpreted when the coloured liquid reached the top of the test and the purple indicator colour had vanished from the filter paper background. As described by the manufacturer, a positive test result was indicated by two purple lines, one in the test zone and the other in the control zone. If a line only appeared in the control zone, the test was considered negative. In cases where only the test line was coloured rather than the control band, the test was declared invalid and was performed once again. The test took approximately 5 to 10 min after deposit of sample and the interpretation was not be performed after 10 min, according to manufacturer recommendations. Following these recommendations, the test was suitable for dog, raccoon dog and cattle samples (animals which were used originally in the validation of the method [18], and should be tested immediately after collection. In this study, two different batches were used for the validation of the RIDT assay, with batches n°1801076 and n°1801111 for the field and the laboratory validation, respectively.

### Extraction of RNA from Anigen test and FTA Whatman cards

Brain impressions were performed directly on FTA Whatman cards, a support dedicated to the storage and preservation of RNA [24]. Prior to use, the cards were stored at room temperature in a sealed plastic bag in a dry and clean area. The samples were prepared by diluting a small section (approximately 0.1 g) of the brain in 1 ml PBS (10%). After thorough mixing, the brain homogenate was loaded onto the card with a pipette until the sample indicator circle on the filter was covered. The cards were then dried 24 hours at room temperature before being put separately in transparent plastic bags for transportation.

To prepare the samples on FTA Whatman filter paper, 1 cm^2^ was cut-off from the area containing the brain impression and incubated during 1 hour in Tri-Reagent LS (Molecular Research Center, Cincinnati, Ohio, USA) or overnight in cell culture medium (DMEM) (Life Technologies, Saint Aubin, France), then placed in Tri-Reagent. To obtain viral RNA directly from the Anigen test strip, the cassettes were opened, the filter paper was removed and the area where the sample was deposited was collected and placed into 1 mL of Tri-Reagent LS. For both FTA and Anigen test supports, total RNA extraction was performed as previously described, following manufacturer recommendations [25].

### Viral RNA detection by RT-qPCR

Viral RNA detection was performed using a one-step dual combine pan-lyssavirus RT-qPCR assay recently described [26], targeting a conserved region among the polymerase. Briefly, this assay includes a pan-RABV RT-qPCR probe-based technique, able to detect all representatives of the broad genetic diversity of RABV, using two degenerated TaqMan probes. In parallel, a SYBR Green RT-qPCR assay is able to detect all the other lyssaviruses tested, in addition to RABV isolates. Both of these assays, which were optimized to a final reaction volume of 20 μL, were performed using 5 μL of RNA template (previously diluted 1:10 in nuclease-free water). For each assay, appropriate controls were used. Details of the combined pan-lyssavirus RT-qPCR assay are in S2 Table and in reference [26].

### Evaluation of viral RNA extracted from Anigen test strips for genotyping

A selected panel of RNA extracts from Anigen test, which were found positive with the dual combine RT-qPCR assay, were evaluated with RT-PCR to generate amplicons suitable for genotyping by sequencing (at least 500 nt in length). Briefly, a volume of 6 μl of total RNA extraction was used for reverse transcription as previously described [25]. RNA was incubated at 65°C for 10 min with 2 μL of pd(N)_6_ random primers (200 μg/mL; Roche Diagnostics) and 2 μL of sterilized distilled water and then were stored on ice. Each tube was incubated with 200 U of Superscript II RT (Invitrogen), 80 U of RNasin (Promega), and 10 nmol of each nucleotide triphosphate (Eurobio), in a final volume of 30 μL for 90 min at 42°C, for reverse transcription. Two microliters of complementary DNA (cDNA) were then amplified by PCR targeting the nucleoprotein gene of RABV, as described in [27].

### Evaluation of the Anigen test in an inter-laboratory trial

The RIDT assay was evaluated by the NRC-R in an inter-laboratory trial organized during 2015 by the European Union reference laboratory for rabies, which is located in Nancy, France [28]. The FAT technique was also evaluated in parallel in this trial. The test panel consisted of nine anonymous samples of freeze-dried homogenized brains, either uninfected or infected with various lyssavirus species. Details of this trial have been provided elsewhere [28].

### Statistical analysis

Results obtained with FAT and Anigen techniques were compared using the McNemar and Kappa statistic tests in Stata, and were analyzed to determine the intrinsic parameters of the RIDT assay. However and conversely to the FAT technique done at the NRC-R, the immunofluorescence assay performed at the IRED could not be considered as the reference technique due to several deviations compared to the standard procedure. In case of discrepancy between RIDT and FAT, samples were tested for RNA detection with RT-qPCR assay performed on FTA Whatman cards impregnated with the brain of the corresponding sample. For the determination of the sensitivity and the specificity of RIDT, true positivity and true negativity was defined according to the result that was shared by at least two tests among FAT, RIDT and viral RNA detection.

## Results

### RIDT test processing and interpretation

**Fig 1:**
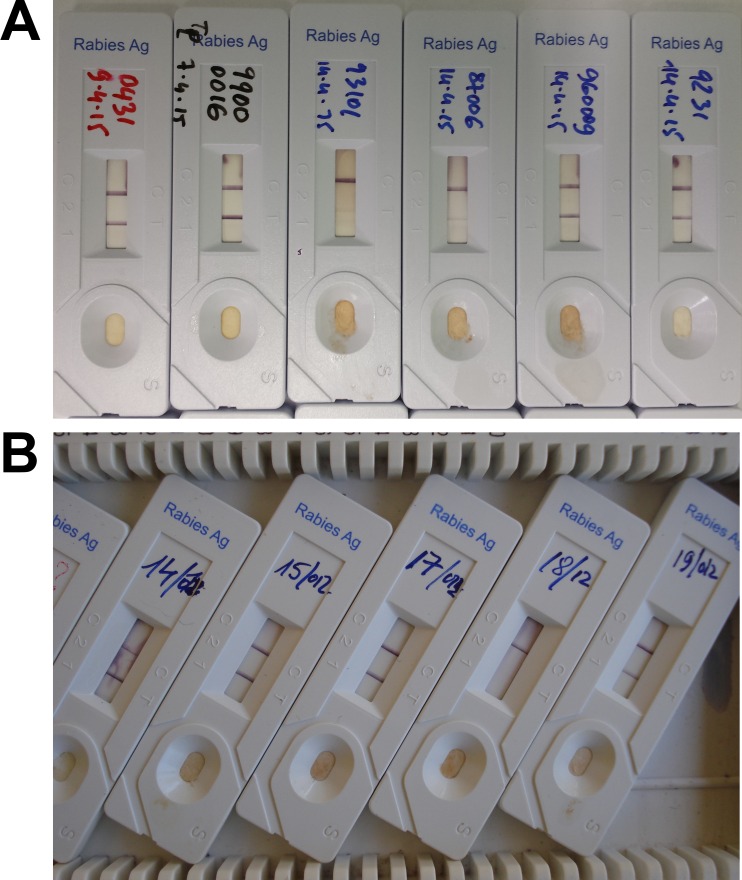
Examples of RIDT results. (A) Results from NRC-R, with six positive results including the two middle cassettes (samples 93101 and 87006) demonstrating only a weak test line in the results window. (B) Results from Chad, with one negative result (sample 18/12) and four positive results. The upper line visible in the results window is the control (C) and the lower line represents the test (T). The presence of two lines depicts a positive result, whereas the presence of only the upper line indicates a negative result. The test is considered as invalid when the upper line is not visible (not shown).

For the majority of the total sample size (n = 121) tested at IRED and at NRC-R, the RIDT was successfully performed, with the presence of a line clearly visible in the control zone after 5 to 15 min of migration once the sample was deposited (Fig 1A and 1B). For only a few samples (n<10), the test was repeated, due to abnormal or incomplete migration (absence of the line in the control zone). When they scored positive with RIDT, most samples exhibited a line with strong intensity in the test zone (Fig 1B). In a few cases the test bands showed even higher intensity than the control band. Also, for some samples tested at NRC-R, the line in the test area was only faintly visible, despite a strong intensity of the line in the control zone (Fig 1A).

### Determination of the limit of detection of the RIDT

A total of eight titrated suspensions from different RABV adapted to cell culture was selected to determine the limit of detection of the RIDT (Table 2). A volume of 100 μL of each of them, diluted or not, were tested. The lowest number of fluorescent focus-forming units (FFU) detected with this assay was 10^5^ FFU, and was obtained for RABV 9704ARG and 04030PHI. Isolates 9147FRA and 9508CZK exhibited a positive signal with 10^6^ FFU. Lastly, no positive signal was obtained with the initial viral suspension for virus 8743THA and 9001FRA, indicating that the limit of detection was > 8.1 x 10^6^ FFU and > 2.4 x 10^5^ FFU, respectively.

**Table 2:** Limit of detection of the RIDT using 8 different titrated rabies virus suspensions.

Virus strain^a^	Original host	Location	Initial virus concentration (FFU/mL)^b^	Limit of detection (FFU)^c^
9147FRA	Red fox	France	3.1 x 10^7^	10^6^
CVS	Lab isolate	-	1.6 x 10^7^	10^6^
8743THA	Human	Thailand	8.1 x 10^7^	> 8.1 x 10^6^
9508CZK (SAD)	Lab isolate	-	5.4 x 10^8^	10^7^
PV	Lab isolate	-	4.3 x 10^7^	10^6^
9001FRA	Dog	French Guiana	2.4 x 10^6^	> 2.4 x 10^5^
9704ARG	Bat	Argentina	9.5 x 10^7^	10^5^
04030PHI	Human	Philippines	2.5 x 10^7^	10^5^

^a^ CVS: Challenge virus strain, SAD: Street Alabama Dufferin, PV: Pasteur virus.

^b^ Number of fluorescent focus-forming units (FFU) per mL.

^c^ Number of fluorescent focus-forming units (FFU) deposited on the strip.

### Comparison results between FAT and RIDT

Seventy-three samples from NRC-R, including forty-three positive samples representing a large diversity in term of host species, geographical location and genetic diversity (S1 and S3 Tables) were tested. Compared to the gold standard FAT, the RIDT demonstrated an accordance of 95%. The specificity was 93.3% with only two false positive results among the 30 FTA-negative specimens, noticed for samples 150057 and 150125 which were originated from a dog and a cat, respectively (Table 3, S1 and S3 Tables). The sensitivity of the RIDT was 95.3%, with only two false negative results observed for isolates 9217ALL and 9312MAU, a red fox from Germany and a dog from Mauritania, respectively (Table 3, S1 and S3 Tables).

**Table 3:** Intrinsic parameters of the RIDT after laboratory evaluation (NRC-R), under field conditions (IRED) and when combining both conditions (all).

			FAT results			
			Pos	Neg	Imp	Total
						
	NRC-R	Pos	41	2	0	43
		Neg	2	28	0	30
		Total	43	30	0	73
						
	IRED	Pos	34	0	0	34
RIDT results		Neg	2	11	1	14
		Total	36	11	1	48
						
	All	Pos	75	2	0	77
		Neg	4	39	1	44
		Total	79	41	1	121

Among the 48 samples included for evaluation at IRED only 3 were not concordant between FAT and RIDT, yielding an accordance of 94% (Table 3). Two of the discordant samples (samples 343 and 389) were decomposed and the quality remained unknown for the last one (sample 362) (Table 1). The FAT was impossible to perform on one (sample 389) of the 3 and for the two others (samples 343 and 362), the result was positive (S4 Table). For all these 3 specimens, RIDT tested negative (S4 Table). In these three cases, where RIDT and FAT did not yield the same result, viral detection performed by RT-qPCR on the FTA Whatman card could not detect viral RNA after multiple attempts confirming the negative RIDT result (S4 Table). For sensitivity and specificity of RIDT and FAT under field conditions, true positivity and true negativity was defined according to the result that was shared by at least two tests among FAT, RIDT and viral RNA detection on the FTA Whatman card. The RIDT showed a higher specificity (100%) than the FAT (78.5%) at IRED. Accordance with the overall true test results of the 48 samples from IRED was 100% for RIDT and 94% for FAT.

The McNemar test showed no significant difference between the FAT and RIDT (Exact McNemar significance probability = 0.5) on samples test at IRED and the Kappa value was 0.86, indicating excellent agreement between the tests. The exact McNemar significance probability for the comparison of FAT and RIDT performed at NRC-R was 1 and the Kappa value was 0.89. Overall, of the 121 samples analysed at NRC-R and IRED, the McNemar significance probability was found to be 0.45 and the Kappa value was 0.87.

### Evaluation of viral RNA detection by RT-qPCR using Anigen test strips and comparison with results obtained using FTA Whatman cards

A total of 51 samples were tested at NRC-R for viral RNA detection using RT-qPCR on the Anigen test strip, which were previously found positive for the *post-mortem* diagnosis of rabies (Table 4, S1 and S4 Tables). The FAT scored also positive for all of these specimens. Among them, 32 originating from IRED were used during the field evaluation of the RIDT, whereas 19 were obtained in NRC-R during the laboratory evaluation. Positive detection was obtained for 26 (81.2%), 18 (94.7%) and 44 (86.3%) samples from IRED, NRC-R and the two combined, respectively (Table 4). In parallel, detection of viral RNA was also performed at NRC-R on FTA Whatman cards for 31 samples, which were positive after analysis with both FAT and RIDT at IRED (S4 Table). In this case, a perfect concordance (100%) was noticed. In addition, viral RNA from 2 and 6 samples found previously negative with RIDT were not detected after RT-qPCR analysis performed on the Anigen test strips and FTA Whatman cards, respectively (Table 4). When compared to the FTA Whatman card, RT-qPCR performed on the Anigen test strip exhibited a sensitivity of 80.6% (Table 5).

**Table 4:** Detection of viral RNA with RT-qPCR on Anigen test strip used in field conditions (IRED), in laboratory conditions (NRC-R) or combined.

		Detection of viral RNA by RT-qPCR on Anigen test strip performed								
		At IRED			At NRC-R			When combined		
		Pos	Neg	Total	Pos	Neg	Total	Pos	Neg	Total
RIDT results	Pos	26	6	32	18	1	19	44	7	51
	Neg	0	3	3	0	0	0	0	3	3
	Total	26	9	35	18	1	19	44	10	54

**Table 5:** Comparison results of the detection of viral RNA with RT-qPCR performed on FTA Whatman cards and Anigen test strips.

		Anigen test strip		
		Pos	Neg	Total
FTA Whatman card	Pos	25	6	31
	Neg	0	2	2
	Total	25	8	33

### Evaluation of the use of viral RNA for genotyping after extraction from Anigen test strips

A limited panel of viral RNA samples extracted from Anigen test strips, which were previously confirmed positive by RT-qPCR, were secondarily tested for genotyping. A total of 14 samples (4 originating from IRED and 10 from NRC-R) were analyzed, among them 13 (93%) provided PCR amplicons (at least 500 nucleotides in length) targeting regions of the nucleoprotein gene commonly used for genotyping after sequencing (S3 and S4 Tables). The only sample found negative (isolate 9702IND) was weakly positive after FAT and RIDT tests, which could then probably explain the absence of PCR amplification.

### Evaluation of the RIDT Anigen test in an inter-laboratory trial

Finally, we evaluated the RIDT Anigen test in an inter-laboratory trial, in parallel of the FAT on nine anonymous samples. The results obtained were concordant with those expected (Table 6) [28]. In particular, we were able to detect three different RABV isolates (strains CVS27 14–10, GS7 and DR627) constituting the panel, as well as 4 other lyssavirus species, including Duvenhage virus (DUVV), European bat lyssavirus 1 (EBLV-1) and 2 (EBLV-2), and Bokeloh bat lyssavirus (BBLV).

**Table 6:** Evaluation of the RIDT Anigen test in NRC-R in an inter-laboratory trial.

Sample	Lyssavirus species^a^	Strain	FAT	RIDT
1	RABV	CVS 27 14–10	Pos	Pos
2	RABV	GS7	Pos	Pos
3	RABV	DR627	Pos	Pos
4	BBLV	127900	Pos	Pos
5	DUVV	DUVV 05–11	Pos	Pos
6	EBLV-1	122938	Pos	Pos
7	EBLV-2	EBL2 RV1787	Pos	Pos
		(1/30)		
8	Negative	-	Neg	Neg
9	Negative	-	Neg	Neg

^a^ RABV: Rabies virus, BBLV: Bokeloh bat lyssavirus, DUVV: Duvenhage virus, EBLV-1: European bat lyssavirus, EBLV-2: European bat lyssavirus 2.

## Discussion

The aim of our study was to evaluate both in the laboratory and under field conditions the RIDT Anigen test in comparison with the FAT, to investigate the intrinsic parameters of this rapid technique, as well as the relevance of its application in sub-Saharan Africa, a region with the highest estimated per capita death rate due to rabies [4] but with poor data reporting situation [29].

We first investigated the limit of detection of this RIDT using serial dilutions of different titrated suspensions of RABV. This value varied among the isolates tested but remaining relatively high, ranked from 10^5^ to 10^7^ FFU.

We then evaluated the use of RIDT for *post-mortem* diagnosis of animal rabies in the laboratory and under field conditions, and compared it to the FAT assay. Our results demonstrate that the lateral flow test performs similar to the FAT. The accordance between RIDT and FAT was high under both conditions (≥94%), with a specificity of the RIDT from 93.3% to 100%. The sensitivity of this technique was also high in laboratory settings, with 95.3%, and approached 100% under field conditions. Our results indicate the high potential of this test for use in the resource challenged African context. Importantly, we show that the intrinsic performance of RIDT under limited laboratory conditions could be higher compared to the FAT test. Conversely to RIDT, several factors could affect negatively FAT results, including storage and quality of the fluorescent conjugate, maintenance of the fluorescence microscope and experience of the reader. However, given the limited sample size, the explanatory power is not overly strong and further evaluation is highly encouraged.

Lastly, we tested the RIDT Anigen test in parallel of the FAT technique in an international inter-laboratory trial organized by the European Union reference laboratory for rabies, Nancy, France and all results were found concordant.

As underlined by the results obtained under field conditions, the advantages of the immunochromatographic test method are manifold. Samples can be analysed one by one proportionate to the diagnostic demand. This is also true for FAT, however, the conjugate used for the FAT can only be stored for a limited time to ensure the quality of the test. Similarly, storage for the reagents is a cause for quality concern for the DRIT. For both DRIT and FAT, negative and positive controls have to be included in the test procedure for standardization, which is not needed for the RIDT [19]. Storage of the RIDT can be done at room temperature and does not require refrigeration, as is necessary for the conjugates used for the DRIT and FAT test. The tests used at IRED were stored at 20°C in an air conditioned room. There are no data on the reliability of the tests stored at temperatures at above 30°C, as would be encountered in many tropical countries.

The FAT depends heavily on the quality of the immunofluorescent conjugate, the maintenance of the fluorescent microscope and also on an experienced technician reading the microscope slides. In contrast, RIDT is a very easy-to-use kit, which does not require a high level of expertise. This technique is simple to perform and to interpret.

For the dog samples tested in Chad, the test had a specificity and sensitivity of 100%. However, we showed the utility of the Anigen test for many different wild and domestic mammalian species. Our results confirm data obtained from previous studies, and suggest that the spectrum of species which could be tested with this RIDT is larger than recommended by the manufacturer (e.g., dogs, cattle and raccoon dogs) [19, 20, 21].

In our study, we mainly focused on RABV species. In particular, we were able to detect RABV isolates belonging to all of the major phylogenetic clades defined previously [30], with the exception of the Africa 3 clade, which was not been tested in our panel. However, positive detection of isolates belonging to this clade has been already demonstrated [20]. In addition, we were also able to detect 4 different other lyssavirus species, in addition to RABV, when evaluated this technique in an inter-laboratory trial. These results are concordant with those obtained in two other previous studies using different lyssavirus species including African and bat-related lyssaviruses (including Lagos bat virus, Mokola virus and Australian bat lyssavirus), demonstrating that it can also be applicable for the detection of non-RABV lyssaviruses [19, 20, 31].

Several RIDT kits for RABV detection were evaluated using brain samples [18, 20, 21, 32, 33]. A very recent study evaluated 6 commercially available RIDT kits in parallel [34]. Sensitivity of the Anigen test in that study was observed to be unsatisfactory [34].

We modified the test procedure by omitting a dilution step and placing the brain sample directly into the buffer vial provided by the test. The advantage of this approach is that the test can be used with no additional material other than that provided in the kit. This change in methodology might explain the better sensitivity of the test and the higher intensity of the test band compared to other studies [19, 22, 35], because the RABV antigen level is higher without a second dilution. Sharma et al. [35] found that the intensity of the test band decreases with dilution.

Autolysis of samples is less a concern for sensitivity of the RIDT compared to the FAT and PCR [17, 19, 36], which is illustrated by our results. Sample storage in glycerol was suggested to interfere with the optimal test performance, affecting the intensity of the test line [19].

The successful detection of RABV RNA from the Anigen test strip in over 86.3% of samples tested is a promising result and highlights the potential use of the kit as a vehicle for sample submission for further confirmatory diagnostic or genotyping analysis. This potential has also been reported by others [34]. However, the sensitivity was lower when compared to the use of the FTA Whatman card, a dedicated support for storage and preservation of nucleic acids.

The price of less than 10 euros is less expensive compared to the cost of performing FAT [19, 22], but still poses an affordability problem in developing countries.

Further validation has to be conducted with RIDT, especially if the results of this test will guide decision making for PEP. We demonstrated that the sensitivity of RIDT, even high, was not complete compared to FAT. To avoid getting false negative results with this technique, we suggest to confirm all negative results using WHO and OIE reference techniques, such as FAT, before excluding RABV infection in diagnostic samples.

An efficient diagnosis method is just part of the entire process of surveillance and control needed to eliminate rabies, as comparable to translation of efficiency of a vaccine, to the ultimate immunity of the target population [37]. Therefore, all components of the surveillance system, in which the test would be promoted and used, have to be strengthened in parallel [38].

### Conclusions

Specificity and sensitivity of the evaluated Anigen test are only slightly reduced compared to the known reference tests for rabies virus detection in brain samples. The results are promising for field use, where the test could help to establish rapid preliminary diagnostic results, which would be further confirmed using WHO and OIE recommended tests at central laboratories. However, we suggest important changes to the test protocol: skip the dilution step of brain biopsy in PBS and perform the brain homogenate with the swab directly into the specimen tube containing 1 ml of assay diluent, both provided in the kit. We also recommend to provide a more precise sketch depicting the brain sampling method. Rapid rabies tests cannot substitute for the current reference tests, but are crucial for the success of rabies surveillance systems in developing countries. Further, we demonstrated here that the test cassettes can be used as a vehicle to ship viral RNA to reference laboratories for further laboratory confirmation of the diagnosis and for epidemiological investigations.

## Supporting Information

S1 Table Description of samples tested at NRC-R, Paris, France. (DOCX)

S2 Table Oligonucleotide sequences of primers and probes used in the combo RT-qPCR (combination of pan-RABV and pan-lyssa RT-qPCR assays) and in the internal control eGFP-based RT-qPCR assay. (DOCX)

S3 Table Comparison results obtained with samples from NRC-R for the *post-mortem* diagnosis of rabies using FAT and RIDT, and for the detection of rabies virus RNA using Anigen test strip as support material. (DOCX)

S4 Table Comparison results obtained with samples from IRED for the *post-mortem* diagnosis of rabies using FAT and RIDT, and for the detection of rabies virus RNA using Anigen test strip and FTA Whatman card as support material. (DOCX)

## References

[1] Jackson AC. Current and future approaches to the therapy of human rabies. Antiviral Res. 2013;99(1):61–7. 10.1016/j.antiviral.2013.01.003 23369672

[2] Fooks AR, Banyard AC, Horton DL, Johnson N, McElhinney LM, Jackson AC. Current status of rabies and prospects for elimination. Lancet 2014 10 11;384(9951):1389–99. 10.1016/S0140-6736(13)62707-5 24828901 PMC7159301

[3] Knobel DL, Cleaveland S, Coleman PG, Fevre EM, Meltzer MI, Miranda ME, et al Re-evaluating the burden of rabies in Africa and Asia. Bull World Health Organ. 2005;83(5):360–8. 15976877 PMC2626230

[4] Hampson K, Coudeville L, Lembo T, Sambo M, Kieffer A, Attlan M, et al Estimating the global burden of endemic canine rabies. PLoS Negl Trop Dis. 2015;9(4):e0003709 10.1371/journal.pntd.0003709 25881058 PMC4400070

[5] Anderson A, Shwiff SA. The Cost of Canine Rabies on Four Continents. Transbound Emerg Dis. 2015 8;62(4):446–52. 10.1111/tbed.12168 24112194

[6] WHO Expert Consultation on Rabies. Second report. World Health Organ Tech Rep Ser. 2013(982):1–139. 24069724

[7] Nel LH. Discrepancies in data reporting for rabies, Africa. Emerg Infect Dis. 2013;19(4):529–33. 10.3201/eid1904.120185 23628197 PMC3647406

[8] Lembo T, Attlan M, Bourhy H, Cleaveland S, Costa P, de Balogh K, et al Renewed global partnerships and redesigned roadmaps for rabies prevention and control. Vet Med Int. 2011;2011:923149 10.4061/2011/923149 21776359 PMC3135331

[9] Mallewa M, Fooks AR, Banda D, Chikungwa P, Mankhambo L, Molyneux E, et al Rabies encephalitis in malaria-endemic area, Malawi, Africa. Emerg Infect Dis. 2007;13(1):136–9. 17370529 10.3201/eid1301.060810 PMC2725806

[10] Klepac P, Metcalf CJE, McLean AR, Hampson K. Towards the endgame and beyond: complexities and challenges for the elimination of infectious diseases. Philos Trans R Soc Lond B Biol Sci. 2013;368(1623). 10.1098/rstb.2012.0137 PMC3720036 23798686

[11] Townsend SE, Lembo T, Cleaveland S, Meslin FX, Miranda ME, Putra AAG, et al Surveillance guidelines for disease elimination: A case study of canine rabies. Comp Immunol Microbiol Infect Dis. 2013;36(3):249–61. 10.1016/j.cimid.2012.10.008 23260376 PMC3693035

[12] Dean D, Abelseth M, Atanasiu P. The fluorescent antibody test. Laboratory techniques in rabies. 1996;4:88–95. 4219510

[13] Bourhy H, Rollin PE, Vincent J, Sureau P. Comparative field evaluation of the fluorescent-antibody test, virus isolation from tissue culture, and enzyme immunodiagnosis for rapid laboratory diagnosis of rabies. J Clin Microbiol. 1989;27(3):519–23. 2654181 10.1128/jcm.27.3.519-523.1989 PMC267350

[14] Dürr S, Mindekem R, Diguimbye C, Niezgoda M, Kuzmin I, Rupprecht CE, et al Rabies diagnosis for developing countries. PLoS Negl Trop Dis. 2008;2(3):e206 10.1371/journal.pntd.0000206 18365035 PMC2268742

[15] Dyer JL, Niezgoda M, Orciari LA, Yager PA, Ellison JA, Rupprecht CE. Evaluation of an indirect rapid immunohistochemistry test for the differentiation of rabies virus variants. J Virol Methods. 2013;190(1):29–33. 23541783 10.1016/j.jviromet.2013.03.009

[16] Sambo M, Lembo T, Cleaveland S, Ferguson HM, Sikana L, Simon C, et al Knowledge, attitudes and practices (KAP) about rabies prevention and control: a community survey in Tanzania. PLoS Negl Trop Dis. 2014;8(12):e3310 10.1371/journal.pntd.0003310 25473834 PMC4256472

[17] McElhinney LM, Marston DA, Brookes SM, Fooks AR. Effects of carcase decomposition on rabies virus infectivity and detection. J Virol Methods. 2014;207:110–3. 10.1016/j.jviromet.2014.06.024 25010791

[18] Kang B, Oh J, Lee C, Park BK, Park Y, Hong K, et al Evaluation of a rapid immunodiagnostic test kit for rabies virus. J Virol Methods. 2007;145(1):30–6. 17628707 10.1016/j.jviromet.2007.05.005

[19] Servat A, Picard-Meyer E, Robardet E, Muzniece Z, Must K, Cliquet F. Evaluation of a Rapid Immunochromatographic Diagnostic Test for the detection of rabies from brain material of European mammals. Biologicals. 2012;40(1):61–6. 10.1016/j.biologicals.2011.12.011 22245544

[20] Markotter W, York D, Sabeta CT, Shumba W, Zulu G, Le Roux K et al Evaluation of a rapid immunodiagnostic test kit for detection of African lyssaviruses from brain material. Onderstepoort J Vet Res. 2009 6;76(2):257–62 20698445 10.4102/ojvr.v76i2.50

[21] Reta T, Teshale S, Deresa A, Ali A, Getahun G. Evaluation of rapid immunodiagnostic test for rabies diagnosis using clinical brain samples in ethiopia. J Vet Sci Med Diagn. 2013;2:2.

[22] Voehl KM, Saturday GA. Evaluation of a rapid immunodiagnostic rabies field surveillance test on samples collected from military operations in Africa, Europe, and the Middle East. US Army Med Dep J. 2014:27–32. 25074599

[23] Anonymous. Anigen Rapid Rabies AG test kit. BioNote. 2010, 1 Available: http://www.bionote.co.kr/File/Upload/2011/02/16/2011-02-16%285%29.pdf

[24] Picard-Meyer E, Barrat J, Cliquet F. Use of filter paper (FTA) technology for sampling, recovery and molecular characterisation of rabies viruses. J Virol Methods. 2007;140(1):174–82. 17157394 10.1016/j.jviromet.2006.11.011

[25] Dacheux L, Reynes JM, Buchy P, Sivuth O, Diop BM, Rousset D, et al A reliable diagnosis of human rabies based on analysis of skin biopsy specimens. Clin Infect Dis. 2008;47(11):1410–7. 10.1086/592969 18937576

[26] Dacheux L, Larrous F, Lavenir R, Lepelletier A, Faouzi A, Troupin C, Nourlil J, Buchy P, Bourhy H. Dual Combined Real-Time Reverse Transcription Polymerase Chain Reaction Assay for the Diagnosis of Lyssavirus Infection. PLoS Negl Trop Dis. 2016 7 5;10(7):e0004812 10.1371/journal.pntd.0004812 27380028 PMC4933377

[27] Bourhy H, Nakouné E, Hall M, Nouvellet P, Lepelletier A, Talbi C, Watier L, Holmes EC, Cauchemez S, Lemey P, Donnelly CA, Rambaut A. Revealing the Micro-scale Signature of Endemic Zoonotic Disease Transmission in an African Urban Setting. PLoS Pathog. 2016 4 8;12(4):e1005525 10.1371/journal.ppat.1005525 27058957 PMC4825935

[28] Robardet E, Picard-Meyer E, Andrieu S, Servat A, Cliquet F. International interlaboratory trials on rabies diagnosis: an overview of results and variation in reference diagnosis techniques (fluorescent antibody test, rabies tissue culture infection test, mouse inoculation test) and molecular biology techniques. J Virol Methods. 2011 10;177(1):15–25. 10.1016/j.jviromet.2011.06.004 21703307

[29] Taylor LH, Knopf L, the Partners for Rabies P. Surveillance of Human Rabies by National Authorities—A Global Survey. Zoonoses Public Health. 2015 11;62(7):543–52. 10.1111/zph.12183 25683444

[30] Bourhy H, Reynes JM, Dunham EJ, Dacheux L, Larrous F, Huong VT, Xu G, Yan J, Miranda ME, Holmes EC. The origin and phylogeography of dog rabies virus. J Gen Virol. 2008 11;89 (Pt 11):2673–81 10.1099/vir.0.2008/003913-0 18931062 PMC3326349

[31] Mshelbwala P, Abdullahi S, Maikai B, Onyiche E, Ogunkoya A. Evaluation of Two Rapid Diagnostic Tests for Rabies Diagnosis under Field and Laboratory Conditions in Nigeria. J Vaccines Vaccin. 2015;6(272):2.

[32] Nishizono A, Khawplod P, Ahmed K, Goto K, Shiota S, Mifune K, et al A simple and rapid immunochromatographic test kit for rabies diagnosis. Microbiol Immunol. 2008;52(4):243–9. 10.1111/j.1348-0421.2008.00031.x 18426399 PMC7168491

[33] Ahmed K, Wimalaratne O, Dahal N, Khawplod P, Nanayakkara S, Rinzin K, et al Evaluation of a Monoclonal Antibody–Based Rapid Immunochromatographic Test for Direct Detection of Rabies Virus in the Brain of Humans and Animals. Am J Trop Med Hyg. 2012;86(4):736–40. 10.4269/ajtmh.2012.11-0332 22492163 PMC3403755

[34] Eggerbauer E, DeBenedictis P, Hoffmann B, Mettenleiter T.C, Schlottau K, Sabeta C.T, Freuling C.M and Müller T. Evaluation of six commercially available rapid immunochromatographic tests for the diagnosis of rabies in brain material. PLoS Negl Trop Dis 2016 6 23;10(6):e0004776 10.1371/journal.pntd.0004776 27336943 PMC4918935

[35] Sharma P, Singh C, Narang D. Comparison of immunochromatographic diagnostic test with heminested reverse transcriptase polymerase chain reaction for detection of rabies virus from brain samples of various species. Vet World. 2015;8(2):135–8. 10.14202/vetworld.2015.135-138 27047061 PMC4774692

[36] Silva S, Katz I, Mori E, Carnieli P, Vieira L, Batista H, et al Biotechnology advances: a perspective on the diagnosis and research of Rabies Virus. Biologicals. 2013;41(4):217–23. 10.1016/j.biologicals.2013.04.002 23683880

[37] Muthiani Y, Traore A, Mauti S, Zinsstag J, Hattendorf J. Low coverage of central point vaccination against dog rabies in Bamako, Mali. Prev Vet Med. 2015;120(2):203–9. 10.1016/j.prevetmed.2015.04.007 25953653

[38] Lembo T, Partners for Rabies P. The blueprint for rabies prevention and control: a novel operational toolkit for rabies elimination. PLoS Negl Trop Dis. 2012;6(2):e1388 10.1371/journal.pntd.0001388 22389727 PMC3289591

