# Supplementary material for: Validation of a Rapid Rabies Diagnostic Tool for Field Surveillance in Developing Countries
Source: PLoS Negl Trop Dis. 2016 Oct 5;10(10):e0005010. doi: 10.1371/journal.pntd.0005010 (PMC5051951; doi:10.1371/journal.pntd.0005010)
Supplement: S1 Table — (DOCX) [file pntd.0005010.s001.docx]

Table S1 : Description of samples tested at NRC-R, Paris, France.

| **Identification** | **Host** | **Country** | **Year** | **Origin^a^** | **Status^b^** | **Phylogenetic clade^c^** |
| --- | --- | --- | --- | --- | --- | --- |
| 150007 | Cat | France | 2015 | P | Negative | - |
| 150036 | Dog | France | 2015 | P | Negative | - |
| 150038 | Cat | France | 2015 | P | Negative | - |
| 150041 | Cat | France | 2015 | P | Negative | - |
| 150042 | Ferret | France | 2015 | P | Negative | - |
| 150043 | Dog | France | 2015 | P | Negative | - |
| 150044 | Dog | France | 2015 | P | Negative | - |
| 150049 | Dog | France | 2015 | P | Negative | - |
| 150050 | Dog | France | 2015 | P | Negative | - |
| 150051 | Dog | France | 2015 | P | Negative | - |
| 150052 | Cat | France | 2015 | P | Negative | - |
| 150053 | Dog | France | 2015 | P | Negative | - |
| 150054 | Cat | France | 2015 | P | Negative | - |
| 150055 | Red fox | France | 2015 | P | Negative | - |
| 150056 | Cat | France | 2015 | P | Negative | - |
| 150057 | Dog | France | 2015 | P | Negative | - |
| 150058 | Dog | France | 2015 | P | Negative | - |
| 150059 | Dog | France | 2015 | P | Negative | - |
| 150060 | Cat | France | 2015 | P | Negative | - |
| 150061 | Cat | France | 2015 | P | Negative | - |
| 150062 | Cat | France | 2015 | P | Negative | - |
| 150119 | Ferret | France | 2015 | P | Negative | - |
| 150125 | Cat | France | 2015 | P | Negative | - |
| 150127 | Cat | France | 2015 | P | Negative | - |
| 150129 | Cat | France | 2015 | P | Negative | - |
| 150132 | Dog | France | 2015 | P | Negative | - |
| 150133 | Dog | France | 2015 | P | Negative | - |
| 150134 | Red fox | France | 2015 | P | Negative | - |
| 150148 | Horse | France | 2015 | P | Negative | - |
| 150230 | Red fox | France | 2015 | P | Negative | - |
| 8670NIG | Human | Nigeria | ? | M | Positive | Africa 2 |
| 8683GRO | Fox | Greenland | 1980 | P | Positive | Arctic-related |
| 8684GRO | Fox | Greenland | 1981 | P | Positive | Arctic-related |
| 8692EGY | Human | Egypt | 1979 | M | Positive | Cosmopolitan (Africa 1 lineage) |
| 8697BEN | Cat | Benin | 1986 | P | Positive | Africa 2 |
| 8706ARS | Fox | Saudi Arabia | 1987 | P | Positive | Cosmopolitan |
| 8801CAM | Dog | Cameroon | 1987 | P | Positive | Africa 2 |
| 8807ETH | Hyena | Ethiopia | 1988 | P | Positive | Cosmopolitan (Africa 1 lineage) |
| 8808ETH | Dog | Ethiopia | 1987 | P | Positive | Cosmopolitan (Africa 1 lineage) |
| 9003CI | Dog | Ivory Coast | 1989 | P | Positive | Africa 2 |
| 9010NIG | Dog | Niger | 1990 | P | Positive | Africa 2 |
| 9021TCH | Dog | Chad | 1990 | P | Positive | Africa 2 |
| 9024GUI | Dog | Guinea | 1990 | P | Positive | Africa 2 |
| 9104USA | Skunk | USA | 1991 | P | Positive | Arctic-related |
| 9115MEX | Dog | Mexico | 1991 | P | Positive | Cosmopolitan |
| 9136MAU | Goat | Mauritania | 1991 | P | Positive | Africa 2 |
| 9141RUS | Polar fox | Russia | 1988-90 | ? | Positive | Arctic-related |
| 9217ALL | Red fox | Germany | 1991 | P | Positive | Cosmopolitan |
| 9218TCH | Dog | Chad | 1992 | P | Positive | Africa 2 |
| 9228CAR | Dog | Central African Republic | 1992 | P | Positive | Africa 2 |
| 9231NAM | Jackal | Namibia | 1992 | P | Positive | Cosmopolitan (Africa 1) |
| 9302SOM | Dog | Somalia | 1993 | P | Positive | Cosmopolitan (Africa 1) |
| 9305SEN | Dog | Senegal | 1992 | P | Positive | Africa 2 |
| 9312MAU | Dog | Mauritania | 1993 | P | Positive | Africa 2 |
| 9319IRA | Jackal | Iran | ? | P | Positive | Cosmopolitan |
| 9391HON | Fox | Hungary | 1993 | P | Positive | Cosmopolitan |
| 93101TUR | Fox | Turkey | 1993 | P | Positive | Cosmopolitan |
| 93105EST | Fox | Estonia | 1993 | P | Positive | Cosmopolitan |
| 93119ZIM | Dog | Zimbabwe | ? | P | Positive | Cosmopolitan (Africa 1) |
| 94289RWA | Dog | Rwanda | 1994 | P | Positive | Africa 2 |
| 9522BRE | Dog | Brazil | 1995 | P | Positive | Cosmopolitan |
| 9547HAV | Dog | Burkina Faso | 1995 | P | Positive | Africa 2 |
| 9609TCH | Dog | Chad | 1996 | P | Positive | Africa 2 |
| 9613TAN | Dog | Tanzania | 1996 | P | Positive | Cosmopolitan (Africa 1) |
| 96178POL | Fox | Poland | 1994 | P | Positive | Cosmopolitan |
| 9702IND | Human | India | 1997 | P | Positive | Indian subcontinent |
| 9705FRA | Bovine | French Guiana | 1997 | P | Positive | Bat |
| 9915BIR | Dog | Myanmar | 1999 | P | Positive | Asia |
| 9916CAM | Dog | Cambodia | 1999 | P | Positive | Asia |
| 02041CHI | Dog | China | 1987 | P | Positive | Asia |
| 02052AFG | Dog | Afghanistan | 2002 | P | Positive | Arctic-related |
| 04031FRA | Dog | France | 2004 | P | Positive | Cosmopolitan (Africa 1) |
| 04033MAD | Human | Madagascar | 2004 | NA | Positive | Cosmopolitan (Africa 1) |

^a^  M : suckling newborn mouse brain sample, P : original primary brain sample, NA: Not available

^b^: Based on FAT results

^c^: According to Bourhy et al., 2008 (Bourhy H, Reynes JM, Dunham EJ, Dacheux L, Larrous F, Huong VT, Xu G, Yan J, Miranda ME, Holmes EC. The origin and phylogeography of dog rabies virus. J Gen Virol. 2008 Nov;89(Pt 11):2673-81)
