# Supplementary material for: Validation of a Rapid Rabies Diagnostic Tool for Field Surveillance in Developing Countries
Source: PLoS Negl Trop Dis. 2016 Oct 5;10(10):e0005010. doi: 10.1371/journal.pntd.0005010 (PMC5051951; doi:10.1371/journal.pntd.0005010)
Supplement: S2 Table — (DOCX) [file pntd.0005010.s002.docx]

Table S2 : Oligonucleotide sequences of primers and probes used in the combo RT-qPCR (combination of pan-RABV and pan-lyssa RT-qPCR assays) and in the internal control eGFP-based RT-qPCR assay.

| **Application** | **Reference** | **Name** | **Type** | **Length** | **Sequence (5’-3’)** | **Sense** | **Position** |
| --- | --- | --- | --- | --- | --- | --- | --- |
| Pan-RABV RT-qPCR assay  (TaqMan^®^-based) | This study | Taq3long | Primer | 22 | ATG AGA AGT GGA AYA AYC ATC A | S | 7273-7294**^a^** |
|  |  | Taq17revlong | Primer | 25 | GAT CTG TCT GAA TAA TAG AYC CAR G | AS | 7390-7414**^a^** |
|  |  | RABV4 | Probe (FAM/TAMRA) | 29 | AAC ACY TGA TCB AGK ACA GAR AAY ACA TC | AS | 7314-7342**^a^** |
|  |  | RABV5 | Probe (FAM/TAMRA) | 32 | AGR GTG TTT TCY AGR ACW CAY GAG TTT TTY CA | S | 7353-7384**^a^** |
| Pan-lyssa RT-qPCR assay  (SYBR^®^ Green-based) | This study | Taq5long | Primer | 23 | TAT GAG AAA TGG AAC AAY CAY CA | S | 7272-7294**^a^** |
|  |  | Taq16revlong | Primer | 25 | GAT TTT TGA AAG AAC TCA TGK GTY C | AS | 7366-7390**^a^** |
| eGFP internal control assay | Hoffmann et al., 2006 | EGFP1F | Primer | 20 | GAC CAC TAC CAG CAG AAC AC | S | 637-656**^b^** |
|  |  | EGFP2R | Primer | 19 | GAA CTC CAG CAG GAC CAT G | AS | 768-750**^b^** |
|  |  | EGFP | Probe (VIC/TAMRA) | 22 | AGC ACC CAG TCC GCC CTG AGC A | S | 703-724**^b^** |

^a^ According to the Pasteur virus (PV) RABV genome sequence (GenBank accession number M13215).

^b^ According to the cloning vector pEGFP-1 sequence (GenBank accession number U55761).

The one-step, probe-based real-time RT-PCR assay (pan-RABV RT-qPCR) was performed with the Superscript III Platinum One-Step RT-qPCR kit (Life Technologies, Saint Aubin, France), as recommended by the manufacturer, with only minor modifications. Real-time PCR, which was optimized for a final reaction volume of 20 μL, was performed with 10 μL 2x Reaction Mix, 1.5 μL nuclease-free water, 1 μL of each primer Taq3long and Taq17revlong (10 μM), 0.4 μL SuperScript^®^ III RT/Platinum^®^ *Taq* Mix, 0.3 μL of each probe RABV4 and RABV5 (10 μM), 0.25 μL MgSO_4_ (50 mM), 0.2 μL RNasin^®^ recombinant ribonuclease inhibitor (Promega, Charbonnieres, France), 0.05 μL ROX^TM^ reference dye and 5 μL RNA template (previously diluted 1:10 in nuclease-free water). Amplification was carried out according to the following program: 1 cycle of heating at 45°C for 15 min and 95°C for 3 min, followed by 40 cycles of 95°C for 15 s and 61°C for 1 min, during which fluorescence values were recorded. All reactions were carried out as technical duplicates in Thermo Scientific 96-well plates (Life Technologies, Saint Aubin, France), with an Applied Biosystems 7500 Real-Time PCR System (Life Technologies, Saint Aubin, France).

For each RT-PCR, a quantification cycle number (C_q_) was determined as the PCR cycle number at which the fluorescence of the reaction exceeded a value considered to be significantly higher than background by the software associated with the Applied Biosystems 7500 Real-Time PCR System (Life Technologies, Saint Aubin, France). The efficiency (E), slope and correlation coefficient (R^2^) were also determined with this software. All reactions were carried out as technical duplicates. A cutoff ≥ 38 was defined for negative results.

The pan-lyssavirus RT-qPCR assay was performed with the SuperScript III Platinum SYBR® Green One-Step qRT, as recommended by the manufacturer (Life Technologies, Saint Aubin, France), with the same minor modifications as indicated for the pan-RABV assay. In particular, this real-time PCR was optimized for a final volume of 20 μL, using the same mixture composition and the same amount of diluted sample. The primers used were Taq5long and Taq16revlong and the probes were replaced with nuclease-free water. Amplification was performed on a similar thermocycler, as follows: 15 minutes at 45°C, 3 minutes at 95°C, followed by 40 cycles of 15 seconds at 95°C and 1 minute at 55°C, during which fluorescence values were recorded. After the 40 cycles of amplification, a melting analysis was carried out to check the product amplified by determining its specific melting temperature (increase 0.01°C/s, 55-95°C). As previously indicated, the efficiency (E), slope and correlation coefficient (R^2^) were also determined with the software associated with Applied Biosystems 7500 Real-Time PCR System (Life Technologies, Saint Aubin, France). All reactions were carried out as technical duplicates. For this assay, a positive reaction was not based on the Cq value but exclusively on the melting temperature (Tm) value and the shape of the melting curve, both compared to positive and negative controls.
