# Supplementary material for: Validation of a Rapid Rabies Diagnostic Tool for Field Surveillance in Developing Countries
Source: PLoS Negl Trop Dis. 2016 Oct 5;10(10):e0005010. doi: 10.1371/journal.pntd.0005010 (PMC5051951; doi:10.1371/journal.pntd.0005010)
Supplement: S3 Table — (DOCX) [file pntd.0005010.s003.docx]

Table S3 : Comparison results obtained with samples from NRC-R for the *post-mortem* diagnosis of rabies using FAT and RIDT, and for the detection of rabies virus RNA using Anigen strip as support material.

| **Sample identification** | **FAT results** | **Anigen results** | **Anigen RNA detection** | **Anigen RNA genotyping** |
| --- | --- | --- | --- | --- |
| 150007 | Neg | Neg | ND | ND |
| 150036 | Neg | Neg | ND | ND |
| 150038 | Neg | Neg | ND | ND |
| 150041 | Neg | Neg | ND | ND |
| 150042 | Neg | Neg | ND | ND |
| 150043 | Neg | Neg | ND | ND |
| 150044 | Neg | Neg | ND | ND |
| 150049 | Neg | Neg | ND | ND |
| 150050 | Neg | Neg | ND | ND |
| 150051 | Neg | Neg | ND | ND |
| 150052 | Neg | Neg | ND | ND |
| 150053 | Neg | Neg | ND | ND |
| 150054 | Neg | Neg | ND | ND |
| 150055 | Neg | Neg | ND | ND |
| 150056 | Neg | Neg | ND | ND |
| 150057 | Neg | Pos | ND | ND |
| 150058 | Neg | Neg | ND | ND |
| 150059 | Neg | Neg | ND | ND |
| 150060 | Neg | Neg | ND | ND |
| 150061 | Neg | Neg | ND | ND |
| 150062 | Neg | Neg | ND | ND |
| 150119 | Neg | Neg | ND | ND |
| 150125 | Neg | Pos | ND | ND |
| 150127 | Neg | Neg | ND | ND |
| 150129 | Neg | Neg | ND | ND |
| 150132 | Neg | Neg | ND | ND |
| 150133 | Neg | Neg | ND | ND |
| 150134 | Neg | Neg | ND | ND |
| 150148 | Neg | Neg | ND | ND |
| 150230 | Neg | Neg | ND | ND |
| 8670NIG | Pos | Pos | ND | ND |
| 8683GRO | Pos | Pos | ND | ND |
| 8684GRO | Pos | Pos | ND | ND |
| 8692EGY | Pos | Pos | ND | ND |
| 8697BEN | Pos | Pos | Pos | Pos |
| 8706ARS | Pos | Pos | Pos | Pos |
| 8801CAM | Pos | Pos | ND | ND |
| 8807GAB | Pos | Pos | Pos | Pos |
| 8808ETH | Pos | Pos | ND | ND |
| 9003CI | Pos | Pos | Pos | Pos |
| 9010NIG | Pos | Pos | ND | ND |
| 9021TCH | Pos | Pos | Pos | Pos |
| 9024GUI | Pos | Pos | Pos | Pos |
| 9104USA | Pos | Pos | ND | ND |
| 9115MEX | Pos | Pos | ND | ND |
| 9136MAU | Pos | Pos | ND | ND |
| 9141RUS | Pos | Pos | ND | ND |
| 9217ALL | Pos | Neg | ND | ND |
| 9218TCH | Pos | Pos | ND | ND |
| 9228CAR | Pos | Pos | ND | ND |
| 9231NAM | Pos | Pos | Pos | ND |
| 9302SOM | Pos | Pos | ND | ND |
| 9305SEN | Pos | Pos | ND | ND |
| 93101TUR | Pos | Pos | ND | ND |
| 93105EST | Pos | Pos | Pos | ND |
| 93119ZIM | Pos | Pos | ND | ND |
| 9312MAU | Pos | Neg | ND | ND |
| 9319IRA | Pos | Pos | Pos | ND |
| 9391HON | Pos | Pos | Pos | ND |
| 94289RWA | Pos | Pos | ND | ND |
| 9522BRE | Pos | Pos | ND | ND |
| 9547BUR | Pos | Pos | ND | ND |
| 9609TCH | Pos | Pos | ND | ND |
| 9613TAN | Pos | Pos | Pos | ND |
| 96178POL | Pos | Pos | Pos | ND |
| 9702IND | Pos | Pos | Pos | Neg |
| 9705FRA | Pos | Pos | Pos | Pos |
| 9915BIR | Pos | Pos | ND | ND |
| 9916CAM | Pos | Pos | Pos | ND |
| 02041CHI | Pos | Pos | Neg | Pos |
| 02052AFG | Pos | Pos | Pos | ND |
| 04031FRA | Pos | Pos | Pos | Pos |
| 04033MAD | Pos | Pos | Pos | ND |

Pos : positive, Neg : negative, ND : not done
