# Supplementary material for: Validation of a Rapid Rabies Diagnostic Tool for Field Surveillance in Developing Countries
Source: PLoS Negl Trop Dis. 2016 Oct 5;10(10):e0005010. doi: 10.1371/journal.pntd.0005010 (PMC5051951; doi:10.1371/journal.pntd.0005010)
Supplement: S4 Table — (DOCX) [file pntd.0005010.s004.docx]

Table S4 : Comparison results obtained with samples from IRED for the *post-mortem* diagnosis of rabies using FAT and RIDT, and for the detection of rabies virus RNA using Anigen strip and FTA Whatman card as support material.

| **Sample identification** | **FAT results** | **Anigen results** | **FTA Whatman card RNA detection** | **Anigen strip RNA detection** | **Anigen strip RNA genotyping** |
| --- | --- | --- | --- | --- | --- |
| 342 | Neg | Neg | Neg | ND | ND |
| 343 | Pos | Neg | Neg | Neg | ND |
| 344 | Pos | Pos | Pos | Neg | ND |
| 345 | Pos | Pos | Pos | Neg | ND |
| 346 | Pos | Pos | Pos | Neg | ND |
| 347 | Pos | Pos | Pos | Neg | ND |
| 348 | Pos | Pos | Pos | Pos | ND |
| 349 | Pos | Pos | Pos | Pos | ND |
| 350 | Pos | Pos | Pos | Pos | ND |
| 352 | Pos | Pos | Pos | Pos | ND |
| 354 | Pos | Pos | Pos | Pos | ND |
| 355 | Pos | Pos | Pos | Pos | ND |
| 356 | Pos | Pos | Pos | Pos | ND |
| 357 | Pos | Pos | Pos | Pos | ND |
| 358 | Pos | Pos | Pos | Neg | ND |
| 359 | Pos | Pos | Pos | Pos | ND |
| 360 | Neg | Neg | Neg | ND | ND |
| 361 | Pos | Pos | Pos | Pos | ND |
| 362 | Pos | Neg | Neg | Neg | ND |
| 363 | Pos | Pos | Pos | Pos | ND |
| 364 | Pos | Pos | Pos | Pos | ND |
| 365 | Neg | Neg | Neg | ND | ND |
| 366 | Pos | Pos | Pos | Pos | ND |
| 367 | Pos | Pos | Pos | Pos | ND |
| 368 | Pos | Pos | Pos | Neg | ND |
| 369 | Pos | Pos | Pos | Pos | ND |
| 371 | Pos | Pos | Pos | Pos | ND |
| 372 | Pos | Pos | Pos | Pos | ND |
| 373 | Pos | Pos | Pos | Pos | ND |
| 379 | Pos | Pos | Pos | Pos | Pos |
| 380 | Neg | Neg | Neg | ND | ND |
| 381 | Pos | Pos | Pos | Pos | Pos |
| 383 | Neg | Neg | ND | ND | ND |
| 384 | Neg | Neg | ND | ND | ND |
| 386 | Neg | Neg | ND | ND | ND |
| 389 | Imp | Neg | Neg | Neg | ND |
| 390 | Neg | Neg | ND | ND | ND |
| 392 | Neg | Neg | ND | ND | ND |
| 393 | Neg | Neg | ND | ND | ND |
| 394 | Pos | Pos | Pos | Pos | Pos |
| 395 | Pos | Pos | Pos | Pos | Pos |
| 400 | Neg | Neg | ND | ND | ND |
| 401 | Pos | Pos | Pos | Pos | ND |
| 403 | Pos | Pos | Pos | Pos | ND |
| 405 | Pos | Pos | Pos | Pos | ND |
| 406 | Pos | Pos | ND | Pos | ND |
| 407 | Pos | Pos | Pos | Pos | ND |
| 408 | Pos | Pos | Pos | Pos | ND |

Pos : positive, Neg : negative, Imp : impossible, ND : not done
